# Dermal White Adipose Tissue (dWAT) Is Regulated by Foxn1 and Hif-1α during the Early Phase of Skin Wound Healing

**DOI:** 10.3390/ijms23010257

**Published:** 2021-12-27

**Authors:** Barbara Gawronska-Kozak, Katarzyna Walendzik, Sylwia Machcinska, Artur Padzik, Marta Kopcewicz, Joanna Wiśniewska

**Affiliations:** 1 Institute of Animal Reproduction and Food Research, Polish Academy of Sciences, 10-748 Olsztyn, Poland; k.walendzik@pan.olsztyn.pl (K.W.); s.machcinska@pan.olsztyn.pl (S.M.); m.kopcewicz@pan.olsztyn.pl (M.K.); j.bukowska@pan.olsztyn.pl (J.W.); 2 Virus Vector Core, Turku Centre for Biotechnology BioCity, 20520 Turku, Finland; padzik.artur@gmail.com

**Keywords:** skin, wound healing, dermal white adipose tissue, Foxn1, Hif-1α

## Abstract

Dermal white adipose tissue (dWAT) is involved in the maintenance of skin homeostasis. However, the studies concerning its molecular regulation are limited. In the present paper, we ask whether the introduction of two transcription factors, Foxn1 and Hif-1α, into the post-wounded skin of Foxn1^−/−^ mice regulates dWAT during wound healing (days 3 and 6). We have chosen lentivirus vectors (LVs) as a tool to deliver Foxn1 and Hif-1α into the post-wounded skin. We documented that combinations of both transgenes reduces the number, size and diameter of dermal adipocytes at the wound bed area. The qRT-PCR analysis of pro-adipogenic genes, revealed that LV-Hif-1α alone, or combined with LV-Foxn1, increases the mRNA expression of *Pparγ*, *Glut 4* and *Fasn* at post-wounding day 6. However, the most spectacular stimulatory effect of Foxn1 and/or Hif-1α was observed for Igf2, the growth factor participating in adipogenic signal transduction. Our data also shows that Foxn1/Hif-1α, at post-wounding day 3, reduces levels of *CD68* and *MIP-1γ* mRNA expression and the percentage of CD68 positive cells in the wound site. In conclusion, the present data are the first to document that Foxn1 and Hif-1α cooperatively (1) regulate dWAT during the proliferative phase of skin wound healing through the Igf2 signaling pathway, and (2) reduce the macrophages content in the wound site.

## 1. Introduction

The skin, the largest organ of the body, creates the wall between the internal organs and the external environment, thereby forming the first barrier that protects against threats, such as injuries or infections. As the most accessible organ for scientific research, it has been studied for decades, and its anatomy and morphology seem to be thoroughly examined and documented. However, the last 20 years of study have brought profound changes in the view of skin structure complexity and, as a consequence, of its operation and function [1,2,3]. First, the dermis, the main part of the skin considered as a uniform structure, appears to be built up by at least two layers: the upper papillary layer, adjacent to the epidermis, and the lower reticular layer [1]. Those layers differ, due to dermal fibroblast heterogeneity and density and extracellular matrix (ECM) composition [2,4]. A study by Wolnicka et al. revealed that the mouse dermis contains cells that express the adipocyte marker FABP4 [5]. Although the classic studies by Chase et al. [6], in the 1950s, demonstrated the synchrony between dermal adipocytes and hair follicle cycling, it was the work of Wojciechowicz et al. [7], focusing on adipocyte development in mouse dorsal skin, that firmly established the presence of adipocyte tissue within the dermis that the authors named the dermal white adipose tissue (dWAT). The dWAT is a type of white adipose tissue and, similar to other fat depots, its content is regulated by the diet and increases in an obesogenic environment in both young and old mice that are fed a high-fat diet (HFD) [8,9]. In old mice, an increase in dWAT, due to 8 weeks of HFD feeding, seems to compensate for age-related skin (fibroblast-rich dermis) thinning [8]. However, dWAT development, functionality and regulatory mechanisms differ from those of other white depots, e.g., inguinal, gonadal or retroperitoneal [10,11,12]. The line of experiments identified dWAT as an important component in skin homeostasis maintenance, that participates in thermoregulation [13], hair follicle cycling [14,15,16] hair growth and pigmentation [17], immune defence and the recovery of wounded skin [12,18,19].

A study by Schmidt and Horsley on mice lacking mature white adipocytes (AZIP mice), showed that dWAT actively participates in the skin wound healing process and is involved in dermal reconstruction through fibroblast recruitment to the wounded area [18]. They also determined that mature adipocytes after skin injury undergo lipolysis to release fatty acids (FAs), which in turn stimulate macrophage infiltration, whereas post-lipolytic adipocytes change their identity to become myofibroblasts. Walendzik et al. demonstrated that the migration of dermal fibroblasts (DFs) from skin explants collected from HFD mice is much more robust than that from mice fed a low-fat diet (LFD), regardless of animal age (young vs. old) [9]. Follow-up analysis of DFs isolated from the skin of young and old mice fed a low-fat diet (LFD) or HFD, revealed differences in their adipogenic potential, particularly related to animal age, with respect to *Zfp423* (determinant of preadipocyte commitment) and *Zfp521* (negative regulator of adipogenesis) [9]. Moreover, it was also shown that the subpopulation of PDGFRα^+^/CD24^+^/Sca1^+^ DFs, recognized as adipocyte progenitors [20], decreased with animal age, particularly in the group fed the LFD [9]. The origin of intradermal adipocytes, during development and during post-wounding skin reconstruction, are still under debate [20,21]. The study by Driskell et al. revealed that DFs and intradermal adipocytes share the common precursor during development [4]. However, after birth, fibroblasts in the upper (papillary) layer of the dermis lose their ability to differentiate into an adipogenic lineage [7]. Guerrero-Juarez et al., using single-cell RNA-sequencing analysis, revealed high heterogeneity of fibroblasts in skin wound, including a large population of cells expressing high levels of Pdgfrα, the potential precursors for new adipocytes [22,23]. Recently, Plikus et al. showed that, during skin wound healing, myofibroblasts (alpha-Sma-Cre lineage-marked cells) can generate adipocytes in skin wounds [23].

The broad spectrum of dWAT function has prompted research on its molecular regulation, particularly through signals derived from the epidermis [9,10,11,12]. Although limited, this research shows that the epidermal Wnt/β-catenin pathway, through the secretion of ligands for the BMP2 and insulin signaling pathways, regulates adipocyte differentiation [10]. IGF2 and BMP2 signaling in the dWAT regulation were further examined, and the results indicated Foxn1, the epidermal transcription factor, as an initiator of adipogenic signaling cascade [9,12,24]. Foxn1, the transcription factor expressed in the epidermis and in the epithelium of the thymus, regulates keratinocyte differentiation/proliferation in the skin, participates in the pigmentation process and is involved in scar-forming healing in wounded skin [25,26,27,28,29,30]. Our recent data showed that Foxn1 inefficiency in mice (Foxn1^+/−^ mice) results in a decrease in susceptibility to diet-induced obesity [12]. Moreover, the skin of Foxn1^+/−^ mice revealed altered levels of the adipogenesis regulators *Pparγ*, *Fapb4*, and *leptin*, compared to their Foxn1^+/+^ counterparts [12]. We also detected that a gradual decrease in Foxn1 availability in vivo (mice: Foxn1^+/+^, Foxn1^+/−^ and Foxn1^−/−^), accompanied the decrease in the expression levels of *Bmp2* and *Igf2* [12].

Hif-1α, a key regulator of antihypoxic responses under hypoxic conditions, is another factor that has essential roles in skin wound healing. Hif-1α has been shown to be involved in all stages of healing of skin wounds, particularly the early events; namely, the inflammatory response and the proliferative phase, including granulation tissue formation and re-epithelialization [31,32,33,34]. Hif-1α has also been broadly examined in white adipose tissues [35,36,37]. The extensive expansion of white adipose tissues due to overnutrition causes chronic hypoxia, which in turn stabilizes Hif-1α. Hif-1α forms a heterodimer with Hif-1β and then binds to the hypoxia response elements (HREs) of downstream genes to regulate their activity. Interestingly, in contrast to the well-known stimulatory effect of Hif-1α on proangiogenic pathways in white adipose tissues, Hif-1α transactivates genes, leading to extracellular matrix component accumulation and, finally, causing fibrosis and inflammatory cell infiltration [35,38]. Our recent data indicate that Foxn1 controls hypoxia-regulated factors [39]. The skin of wounded and unwounded Foxn1^−/−^ mice displayed low levels of Hif-1α expression that were unchanged by wounding, whereas Hif-1α expression was modulated in the wounded skin of Foxn1^+/+^ mice [39]. In an in vitro study, the overexpression of Foxn1 led to the downregulation of *Hif-1α* mRNA at the expense of the upregulation of *Fih-1* (factor inhibiting Hif-1α), indicating the possible mechanisms by which Foxn1 regulates hypoxia-related genes [39].

Combining the proven role of Hif-1α in skin wound healing and in white adipose tissue regulation with its interaction with Foxn1, in this study, we asked whether the introduction of Foxn1 and/or Hif-1α into the skin of Foxn1^−/−^ mice regulates dWAT during the proliferative phase of wound healing. Considering the easy accessibility of hairless skin, due to Foxn1 deficiency in mice (Foxn1^−/−^; nude mice), we chose lentivirus (LV) as a tool to deliver Foxn1 and/or Hif-1α into the skin. The efficiency and effectiveness of lentivirus vector transgene delivery into skin was proven earlier [40,41,42].

## 2. Results

### 2.1. Lentivirus Is an Effective Vector in Intradermal Foxn1 and Hif-1α Transgene Delivery

**Figure 1:**
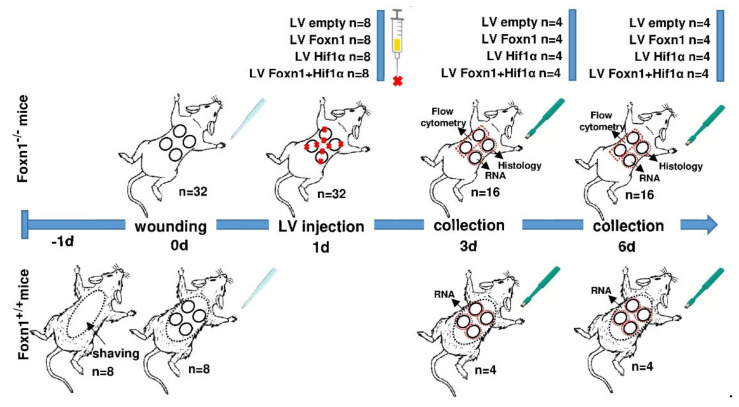
Scheme of the experimental design. Foxn1^−/−^ (nude mice; CBy. Cg-Foxn1 <nu>/cmdb; *n* = 32) and Foxn1^+/+^ (Balb/c) mice of a similar age (*n* = 8) were used. Mice were injured on day 0. Lentivirus vectors (LVs) carrying LV-GFP (empty) or Foxn1+mCherry, or the Hif-1α+eGFP transgene were injected intradermally into the skin of Foxn1^−/−^ mice at day 1. Skin tissues were collected on days 3 and 6, post-wounding; *n* = 4 per time point/per group.

**Figure 2:**
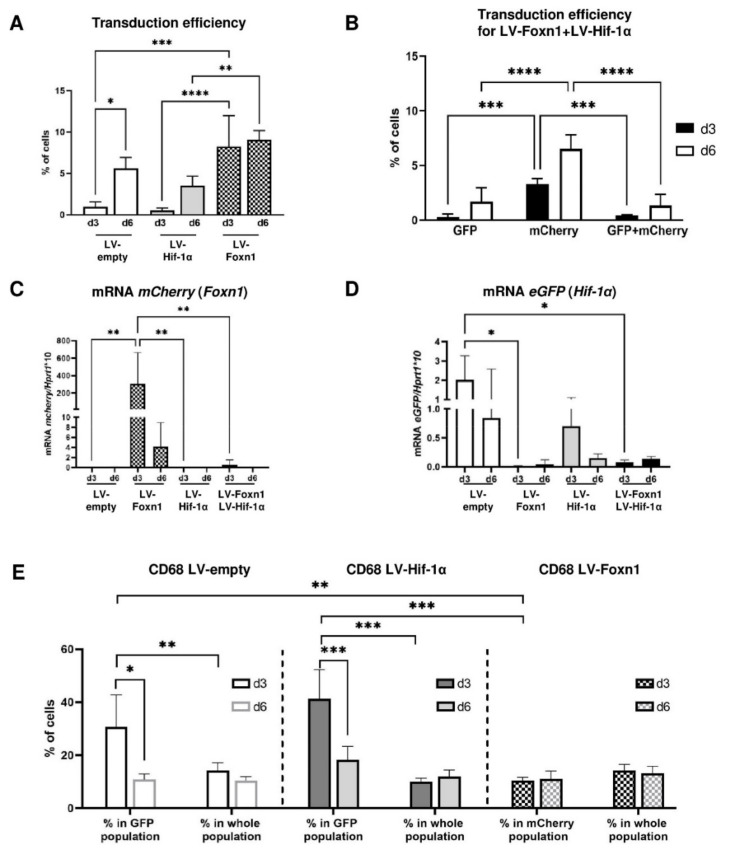
Transduction efficiency of lentivirus vectors (LVs) carrying Foxn1-mCherry, Hif-1α-eGFP or eGFP (empty) injected into the wounded skin of Foxn1^−/−^ mice. Flow cytometry (**A**,**B**,**E**) analyses of cells isolated from the wounded skin at day 3 or day 6 (*n* = 4 per time point/per group). Percentage of cells positive for eGFP or mCherry, isolated from the post-injured skin of (**A**) LV-empty-eGFP, LV-Hif-1α-eGFP or LV-Foxn1-mCherry; (**B**) LV-Hif-1α-eGFP+LV-Foxn1-mCherry injected Foxn1^−/−^ mice; mRNA expression of *mCherry* (**C**) or *eGFP* (**D**) in injured skin on days 3 or 6, collected from LV-empty-eGFP-, LV-Hif-1α-eGFP, LV-Foxn1-mCherry- or LV-Hif-1α-eGFP+LV-Foxn1-mCherry-injected mice. Percentage of CD68-positive cells within eGFP-positive (LV-empty or LV-Hif-1α) or mCherry-positive (LV-Foxn1) populations, in comparison to the whole cell population (**E**). Asterisks indicate significant differences (* *p* < 0.05; ** *p* < 0.01; *** *p* < 0.001; **** *p* < 0.0001). Data represents mean ± SD.

As a first step in the investigation of the potential role of two transcription factors, Foxn1 and/or Hif-1α, in dWAT regulation during the skin wound healing process, we estimated the transduction efficiency of LVs carrying the Foxn1 or Hif-1α transgene. To determine the relative in vivo transduction efficiency, the skin of externally wounded (day 0) and LV-injected (day 1) Foxn1^−/−^ mice was collected post mortem, on days 3 and 6 post-wounding (Figure 1). Skin samples (*n* = 4 per group/per day), each including two circular post-wounding areas, were collected (Figure 1), the cells were isolated and flow cytometry analyses were performed (Figure 2A,B,E; Figures S1–S4). To avoid the possible adverse effect of pH on the fluorescence effectiveness of eGFP or mCherry tagged to Foxn1 (mCherry) or Hif-1α (eGFP), we used antibodies against the eGFP or mCherry protein for flow cytometry assay to estimate the percentage of transduced skin cells [43].

Generally, in cell isolates, we observed an increase in the percentage of cells expressing eGFP (LV-empty, LV-Hif-1α) or mCherry (LV-Foxn1) (Figure 2A,B, Figure S1), or expressing mCherry+eGFP (LV-Foxn1+ LV-Hif-1α; Figure 2B, Figure S1) between the skin samples collected at post-wounding day 3 and at day 6. At day 6, after wounding, eGFP was expressed in 5.58% ± 1.34 of cells isolated from LV-empty injected skin and 4.5% ± 1.16 of cells isolated from LV-Hif-1α skin, whereas mCherry was detected in 9.05% ± 1.11 of cells isolated from the skin of mice injected with LV-Foxn1 (Figure 2A, Figure S1). The analysis of skin samples collected at post-wounding day 6 from mice injected with both LV-Foxn1+ LV-Hif-1α, showed not only an increase in the percentage of cells carrying the Hif-1α (eGFP; 1.7% ± 1.26) or Foxn1 (mCherry; 6.5% ± 1.30) transgene, but also cells (1.35% ± 1.02) that colocalized with both transgenes (Figure 2B, Figure S1).

LVs transduction was also evidenced by the mRNA expression of *mCherry* or *eGFP* in wounded skin (Figure 2C,D). Interestingly, mRNA expression of both *eGFP* and *mCherry* analyzed in the post-injection skin tissues decreased from day 3 to day 6 (Figure 2C,D), which was opposite to the detected increases in eGFP and mCherry protein between days 3 and 6 (percentage of positive cells: Figure 2A,B, Figure S1). Phenotypic analysis/characteristic of cells collected from the skin of LV-injected mice were presented in the supplementary data and Figures S2 and S3).

Next, we analyzed the distribution of CD68-positive cells (a marker of macrophages) within the population of cells carrying transgenes (Hif-1α or Foxn1) with specific reporters (eGFP or mCherry), in relation to the total population of CD68-positive cells collected from injured skin on days 3 and 6 (Figure 2E, Figure S4A,B). In LV-empty (eGFP)-injected skin, the eGFP-positive population contained 30.6% ± 12.10 and 10.8% ± 2.10 CD68-positive cells (days 3 and 6, respectively), whereas the total cell population included 14.2% ± 2.90 and 10.3% ± 1.52 CD68-positive cells (Figure 2E, Figure S4). The analysis of skin tissues collected from LV-Hif-1α-injected mice, showed an increase in CD68-positive cells within the eGFP-positive population: 41.2% ± 11.02 and 18.3% ± 5.03, in comparison to the 9.9% ± 1.37 and 11.9% ± 2.41 in the whole population of cells or to skin injected with control vector on days 3 and 6, respectively (Figure 2E, Figure S4). Interestingly, the distribution of CD68-positive cells did not differ within the LV-Foxn1-injected skin (mCherry-positive population; 10.5% ± 1.18 and 11.1% ± 2.98 on days 3 and 6, respectively) or the whole population (14.2% ± 2.31 and 13.2% ± 2.61 on days 3 and 6, respectively) (Figure 2E, Figure S4).

These data showed the effectiveness of Foxn1 and Hif-1α transgene delivery via LVs injections into the wounded skin of Foxn1^−/−^ mice. It also indicates the affinity of the transgenes carried by LVs to be incorporated into cells with native gene expression, i.e., Foxn1 incorporation was predominantly stimulated by wound keratinocytes (CK6-positive cells; Figures S2 and S3) and dermal fibroblasts (vimentin-positive cells; Figures S2 and S3), whereas Hif-1α was stimulated by keratinocytes (E-cadherin-positive; Figures S2 and S3), dermal fibroblasts (vimentin-positive; Figures S2 and S3) and macrophages (CD68-positive; Figure 2E and Figure S4).

### 2.2. Microscopic Evaluation of Skin Wound Healing

Experimental mice were observed every day for the skin wound healing process and potential adverse effects of the LV injection. No changes in the animals’ activity or behavior, rhythm of breathing or color of feces or urine in the cages were detected during the entire experiment (personal observations).

**Figure 3:**
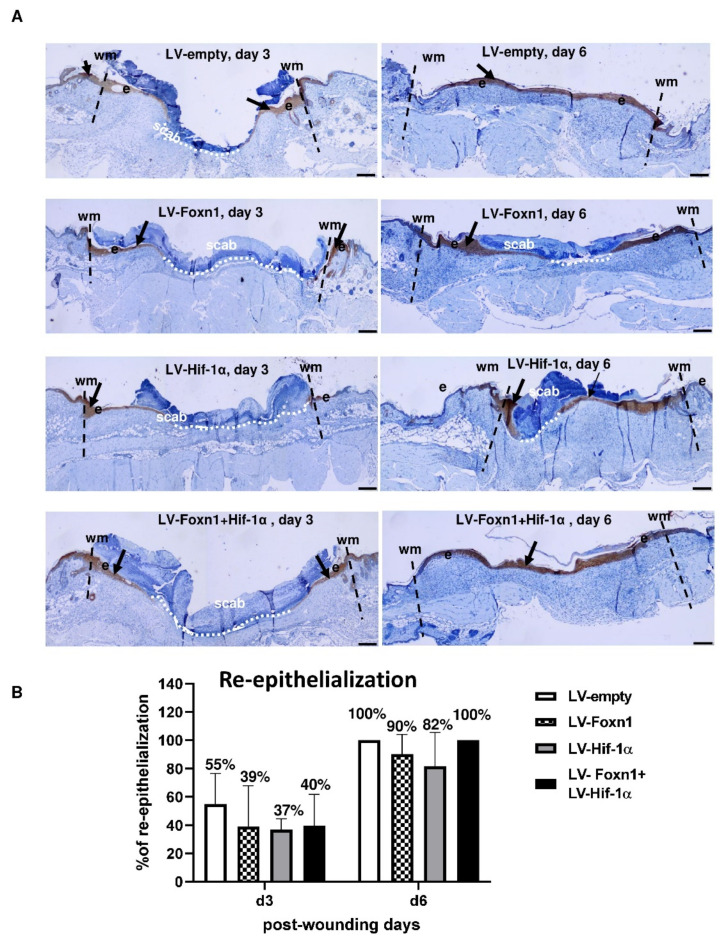
Microscopic evaluation of the re-epithelialization process in LV-empty-, LV-Hif-1α, LV-Foxn1- or LV-Hif-1α+LV-Foxn1-injected mice. (**A**) Representative histological sections of wounded skin stained for keratin 16 positivity (arrows); wm-wound margin, e-epidermis, scale bar 200 μm. (**B**) Morphometrical analysis of the re-epithelization process.

The re-epithelialization was analyzed on histological sections immunostained for cytokeratin 16, the marker of this process (Figure 3). On day 3, the fastest pace of re-epithelization was observed in LV-empty-treated mice (55% of wound cover), and the slowest pace was observed in skin injected with LV-Hif-1α (37%) (Figure 3). The process of re-epithelialization was completed on day 6 for LV-empty/control- and LV-Foxn1+LV-Hif-1α-treated mice (100%; Figure 3). Our previous study showed 94% of re-epithelialization on post-wounding day 3, and completion of the process on day 5 during skin wound healing of Foxn1^−/−^ mice [39]. Observed discrepancies in the re-epithelialization pace between previous and present experiments are most likely caused by LVs injection, per se. 

The analysis of post-wounded skin re-establishment showed the fastest rate of re-epithelialization for LV-empty-injected Foxn1^−/−^ mice, although the difference did not reach a statistical significance (Figure 3B).

### 2.3. Combined Effect of Foxn1 and Hif-1α on Dermal Adipocyte (dWAT) Morphology

**Figure 4:**
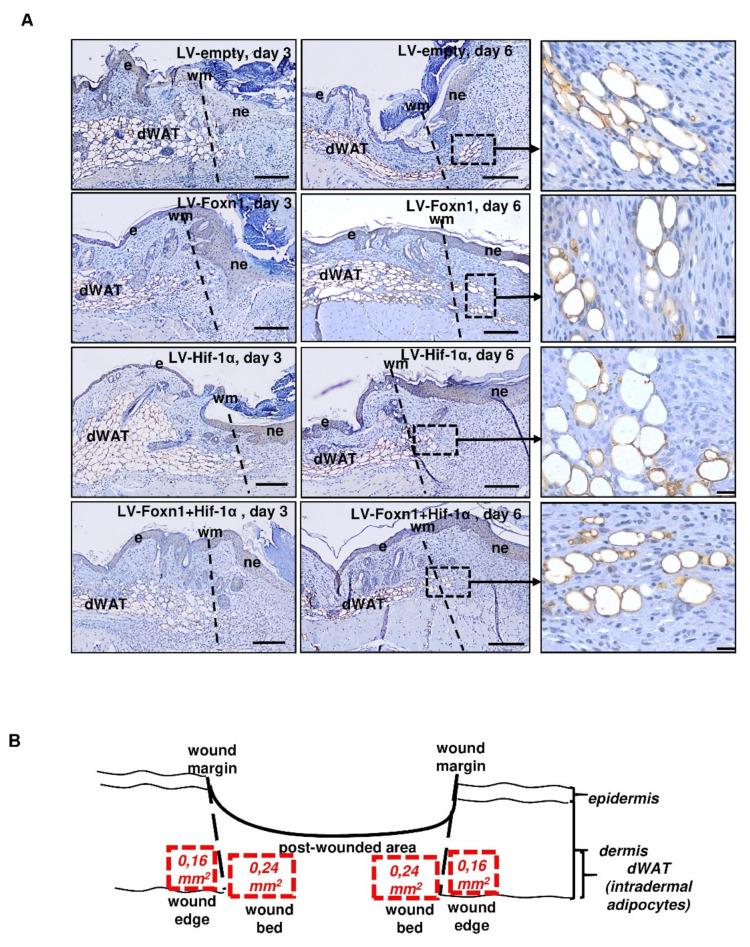
Immunohistochemical detection of perilipin 1 (**A**) as a marker of dWAT presence at the wound edge and wound bed (**B**) in LV-empty-, LV-Hif-1α-, LV-Foxn1- or LV-Hif-1α+LV-Foxn1-injected mice analyzed on days 3 and 6, post-wounding (*n* = 3–4 mice per group/per day). (**B**) the scheme indicates the area (red squares) of wound edge and wound bed of perillipin 1 positive dWAT analysis; e—epidermis, ne—neo-epidermis, wm—wound margin, and dWAT—dermal white adipose tissue, scale bar (**A**) 100 μm, inset 20 µm.

**Figure 5:**
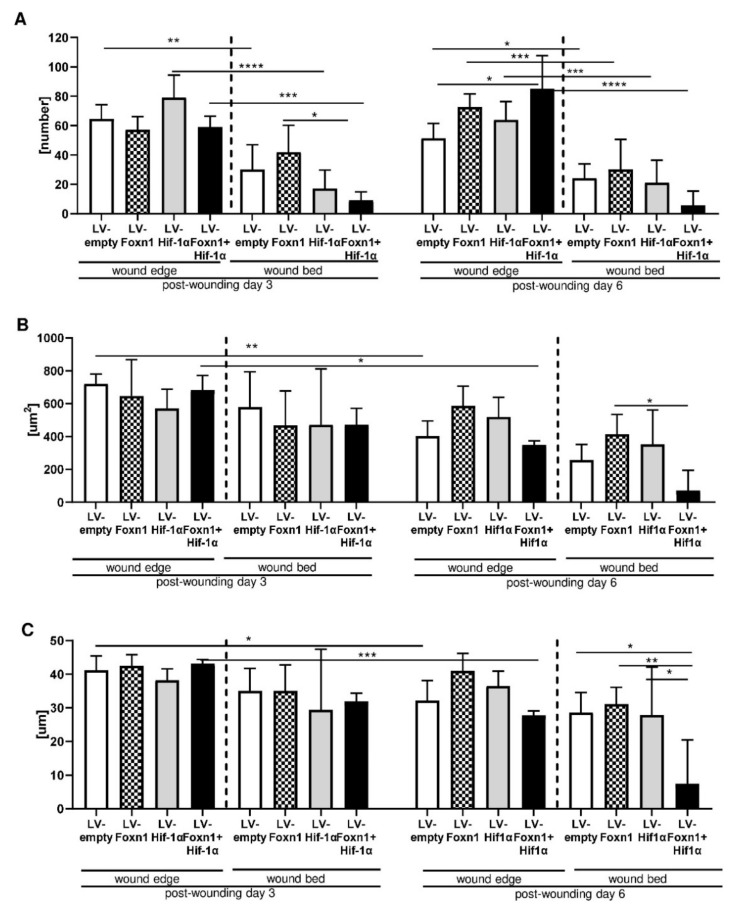
Quantitative analysis of adipocytes: number (**A**), size (**B**) and diameter (**C**) based on perilipin 1-stained skin sections (see Figure 5A) from LV-empty, LV-Hif-1α, LV-Foxn1- or LV-Hif-1α+LV-Foxn1-injected mice. Values are the mean ± SD. Asterisks indicate significant differences (* *p* < 0.05; ** *p* < 0.01; *** *p* < 0.001; **** *p* < 0.0001).

Having established the effectiveness of Foxn1 and Hif-1α transgene delivery and functionality, we analyzed dWAT in the skin (Figure 4 and Figure 5). Post-wounding skin sections collected from Foxn1^−/−^ mice that were injected with LV-Foxn1, LV-Hif-1α, LV-Foxn1+LV-Hif-1α or control (LV-empty), were stained for the presence of perilipin 1, a protein that localizes on lipid droplets and serves as a marker of adipocytes (Figure 4). The adipocyte number (Figure 5A), size (Figure 5B) and diameter (Figure 5C) were examined at the wound edge and wound bed, separately (Figure 4B).

At the wound edge area (Figure 4A,B) on post-wounding day 3, we did not observe statistically significant differences in the adipocyte number (Figure 5A), size (Figure 5B) or diameter (Figure 5C), among the LV-Foxn1, LV-Hif-1α, LV-Foxn1 +LV-Hif-1α- or LV-empty-treated groups. On post-wounding day 6, e.g., 5 days after transgene delivery, an increase in the adipocyte number at the wound edge was detected in LV-Foxn1+LV-Hif-1α-treated skin (Figure 5A), and an increase in adipocyte size (Figure 5B) and diameter (Figure 5C) was detected in LV-Foxn1-treated mice. Although the increases in adipocyte size and diameter were observed in mice injected with LV-Foxn1, the injection of combined transgenes (Foxn1 + Hif-1α) resulted in unchanged dWAT morphology, similar to that in the control (LV-empty) (Figure 5B,C). We also observed a decrease in adipocyte size and diameter at the wound edge area, from days 3 to day 6 after wounding, for LV-Foxn1+LV-Hif-1α and control (LV-empty) skin (Figure 5B,C).

The wound bed area was affected by transgene delivery on post-wounding day 3, when the number of adipocytes was the smallest for LV-Foxn1+LV-Hif-1α-treated skin (Figure 5A). On day 6, the combined effects of LV-Foxn1+LV-Hif-1α in decreasing the adipocyte number, size and diameter at the wound bed were further observed (Figure 5A–C). As observed at the wound edge, in the wound bed, the delivery of both transgenes, LV-Foxn1+LV-Hif-1α, but not the individual delivery of LV-Foxn1 or LV-Hif-1α profoundly decreased the adipocyte number, size and diameter (Figure 5A–C).

These data indicate that the combined action of both Foxn1 and Hif-1α has the greatest effect on the characteristics of post-wounding dWAT.

### 2.4. Foxn1 and Hif-1α Cooperatively Regulate dWAT during the Proliferative Phase of Skin Wound Healing through the Igf2 Signaling Pathway

The apparent differences in the adipocyte number and adipocyte size detected among treated groups, particularly in the wound bed area, the site of new tissue formation, can suggest not only differences in lipid content/lipolysis, as shown by Shook et al. [19], but also relates to Foxn1 and/or Hif-1α as factors in the skin that regulate posttraumatic adipogenesis.

**Figure 6:**
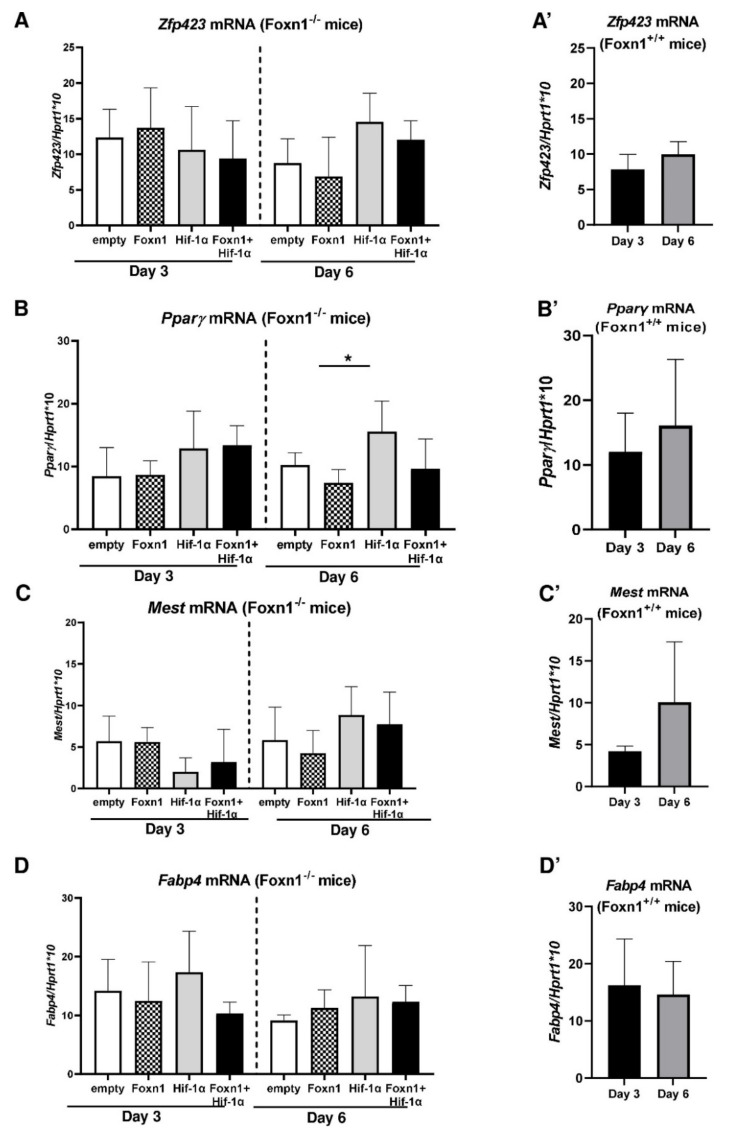

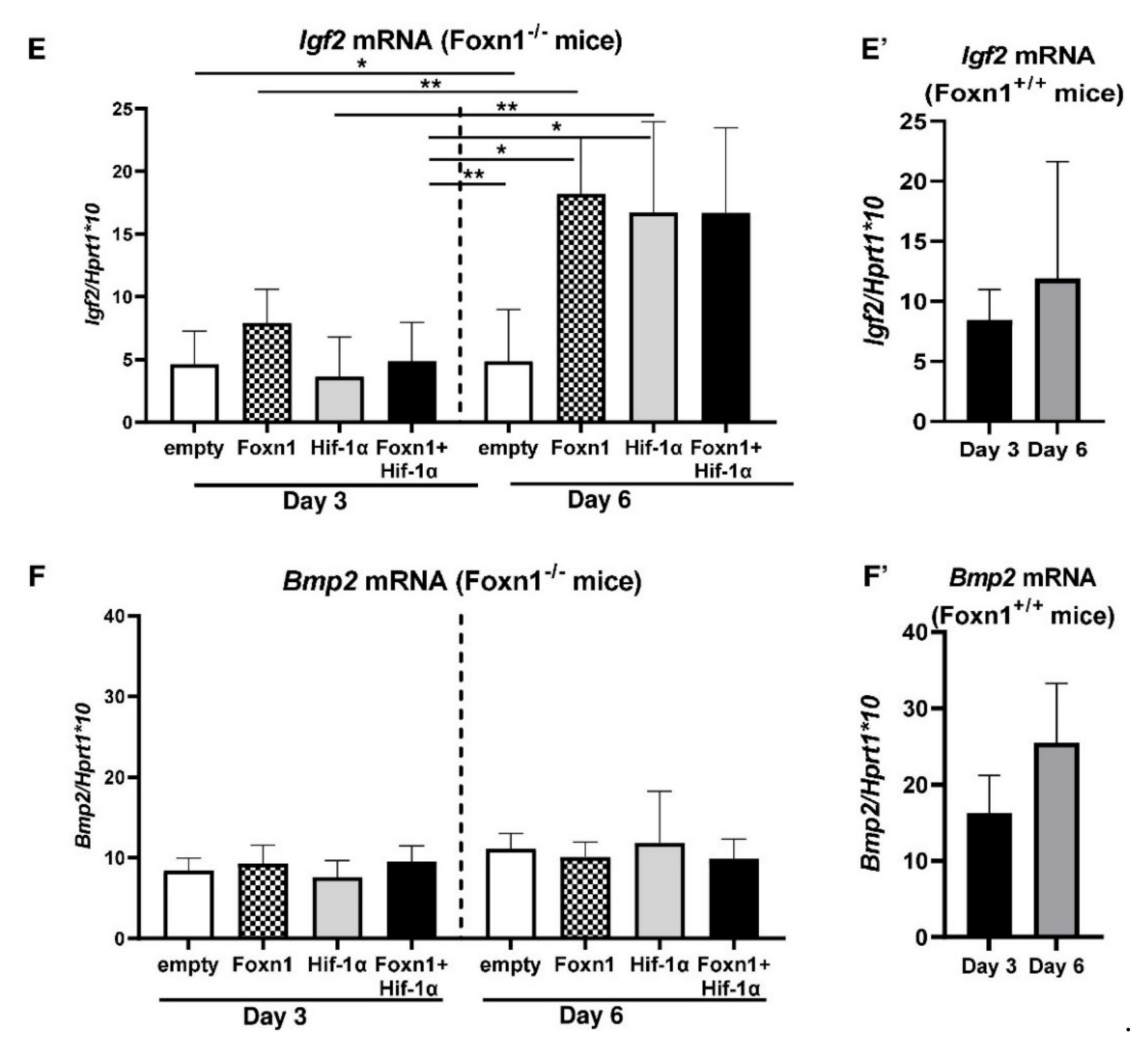
Quantitative RT-PCR mRNA expression analysis of adipogenic-related genes in the skin of Foxn1^−/−^ mice injected with LV-empty, LV-Hif-1α, LV-Foxn1 or LV-Hif-1α+LV-Foxn1 (**A**–**F**) and Foxn1^+/+^ (Balb/c) mice (**A’**–**F’**): *Zfp423* (**A**,**A’**); *Pparγ* (**B**,**B’**); *Mest* (**C**,**C’**); *Fabp4* (**D**,**D’**); *Igf2* (**E**,**E’**); and *Bmp2* (**F**,**F’**). Values are the mean ± SD, *n* = 4 mice per group/per time point. Asterisks indicate significant differences (* *p* < 0.05; ** *p* < 0.01).

For the assessment of the potential role of Hif-1α and Foxn1 in skin adipogenesis, we analyzed the expression of molecular regulators of adipogenesis: *Pparγ*, the master regulator of adipogenesis; *Fabp4*, an indicator of differentiated adipocytes; *Mest*, a mesoderm-specific transcript, as a marker of expansion and size of the adipocytes; *Zfp423*, zinc finger transcription factor recognized as regulator of adipose commitment; and *Bmp2* and *Igf2,* two growth factors recognized as adipogenic signal transduction pathways, in the skin of mice treated with LVs carrying transgenes (Figure 6A–F). We also analyzed the expression of the same adipogenic factors in the corresponding skin tissues from Foxn1^+/+^ (Balb/c) mice (Figure 6A’ and Figure 8F’). Since Foxn1^+/+^ mice did not receive the same treatment as Foxn1^−/−^ mice, i.e., LV injection, we did not perform a comparison analysis between them, considering Foxn1^+/+^ mice as a separate, noncomparable entity. However, we applied the same scale in the presented graphs (compare Figure 6A,A’; Figure 6B,B’; Figure 6C,C’; Figure 6D,D’; Figure 6E,E’; and Figure 6F,F’) to observe at least approximate differences/similarities in gene expression between LV-treated (Foxn1^−/−^) and non-LV-treated (Foxn1^+/+^) mice.

There were no statistically significant differences in *Zfp423*, *Pparγ*, *Fabp4* and *Mest* mRNA expression among the treated groups on day 3 post-wounding, although a decrease in *Zfp423* mRNA and an increase in *Pparγ* mRNA were observed in the LV-Hif-1α- and LV-Hif-1α+LV-Foxn1-treated groups in comparison to the LV-Foxn1- and control groups. (Figure 6A–D). Further differences were detected on post-wounding day 6. The injection of LV-Hif-1α alone in the skin displayed the most evident effect, manifested by the increase in the mRNA expression of all adipogenic markers: *Zfp423*, *Pparγ* (*p* < 0.05), *Fabp4* and *Mest* (Figure 6A–D). Interestingly, the combined effects of LV-Foxn1+LV-Hif-1α on *Zfp423*, *Pparγ*, *Fabp4* and *Mest* mRNA seemed to reduce the effect induced by LV-Hif-1α treatment alone (Figure 6A–D).

Next, we examined the expression of *Bmp2* and *Igf2* mRNA levels, the two growth factors recognized as adipogenic signal transduction pathways, in the skin of mice treated with LVs carrying transgenes (Figure 6E,F). On post-wounding day 3, an increase in *Igf2* mRNA expression was observed solely in LV-Foxn1-treated skin (Figure 6E). Interestingly, the levels of *Igf2* mRNA expression achieved after LV-Foxn1 injection into the skin of Foxn1^−/−^ mice were similar to the levels observed in the skin of Foxn1^+/+^ mice (compare Figure 6E,E’). The differences were further observed on post-wounding day 6. A robust increase in *Igf2* mRNA expression was detected in all treated groups, LV-Foxn1 (*p* < 0.01), LV-Hif-1α (*p* < 0.05) and LV-Foxn1+LV-Hif-1α (*p* < 0.05), in comparison to the skin of control (LV-empty)-treated Foxn1^−/−^ mice (Figure 6E). Moreover, this increase was statistically significant between days 3 and 6 within the LV-Foxn1 (*p* < 0.05), LV-Hif-1α (*p* < 0.01) and LV-Foxn1+LV-Hif-1α (*p* < 0.01) groups, but not in the control (LV-empty) group (Figure 6E). In contrast, *Bmp2* mRNA expression was unaffected by transgene delivery and did not change between days 3 and 6 after wounding in any of the groups (Figure 6F). We also did not observe differences in *Bmp2* mRNA expression between Foxn1^−/−^ mice (regardless of treatment) and Foxn1^+/+^ mice (compare Figure 6F,F’).

**Figure 7:**
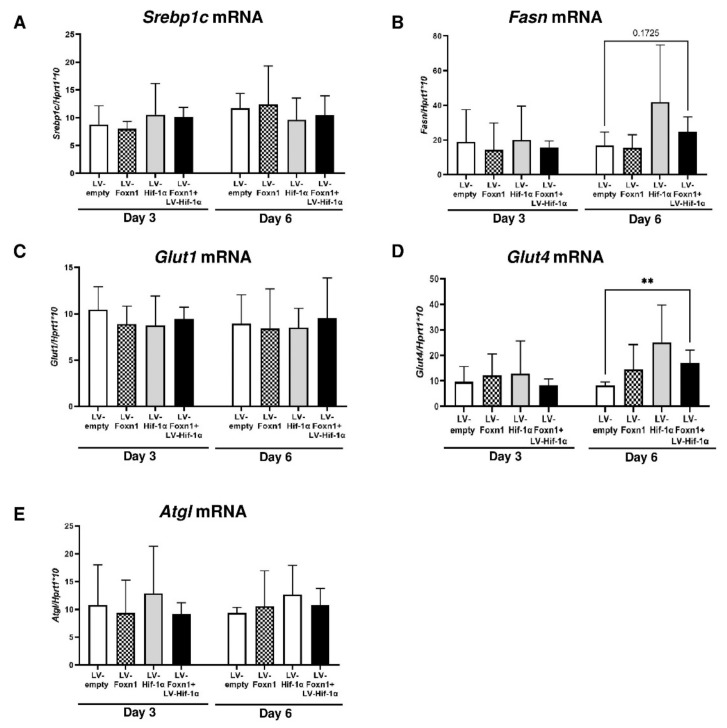
Quantitative RT-PCR mRNA expression analysis of genes related to lipogenesis: *Srebp1c* (**A**), *Fasn* (**B**), *Glut1* (**C**) and *Glut4* (**D**), or lipolysis: *Atgl* (**E**). Data represents mean ± SD, *n* = 4 mice per group/per time point (** *p* < 0.01).

To further evaluate the possible impact of Foxn1/Hif-1α on dWAT functional reconstruction after skin injury, we analyzed the expression of genes involved in de novo lipogenesis (*Srebp1c*—sterol regulatory element-binding protein 1c, *Fasn*—fatty acid synthase and *Glut1* and *Glut4*—transmembrane glucose transporters; Figure 7A–D) and lipolysis (*Atgl*—adipose triglyceride lipase; Figure 7E).

There were no differences in the expression of lipogenic genes *Srebp1c*, *Fasn*, *Glut1* and *Glut4*, among treated groups on post-wounding day 3 (Figure 7A–D). On post-wounding day 6, skin tissues treated with LV-Hif-1α and LV-Foxn1+LV-Hif-1α showed an increase in the levels of *Fasn* mRNA expression, although the observed increase did not reach statistical significance (Figure 7B). We detected, however, significant differences in the glucose transporter 4 mRNA expression levels (Figure 7C,D). Whereas *Glut1* mRNA was unchanged and showed similar levels of expression among treatment groups and between post-wounding days (day 3 vs. day 6; Figure 7C), *Glut4* expression, particularly at day 6, was affected by the treatment (Figure 7D). Combined LV-Foxn1 and LV-Hif-1α delivery, increased the levels of *Glut4* mRNA expression comparing to LV-empty (control) treated skin (Figure 7D), which corresponds to the increase in *Fasn* mRNA levels (compare Figure 7B,D). The levels of *Atgl* (the lipolysis gene) mRNA expression, was unaffected by treatment, regardless of post-wounding day 3 or 6 (Figure 7E).

In summary, these data support that Foxn1 and Hif-1α, in combination with the Igf2 signaling pathway, can regulate dWAT during the proliferative phase of skin wound healing.

### 2.5. Foxn1 Reduces Macrophage Content in Wounded Skin

**Figure 8:**
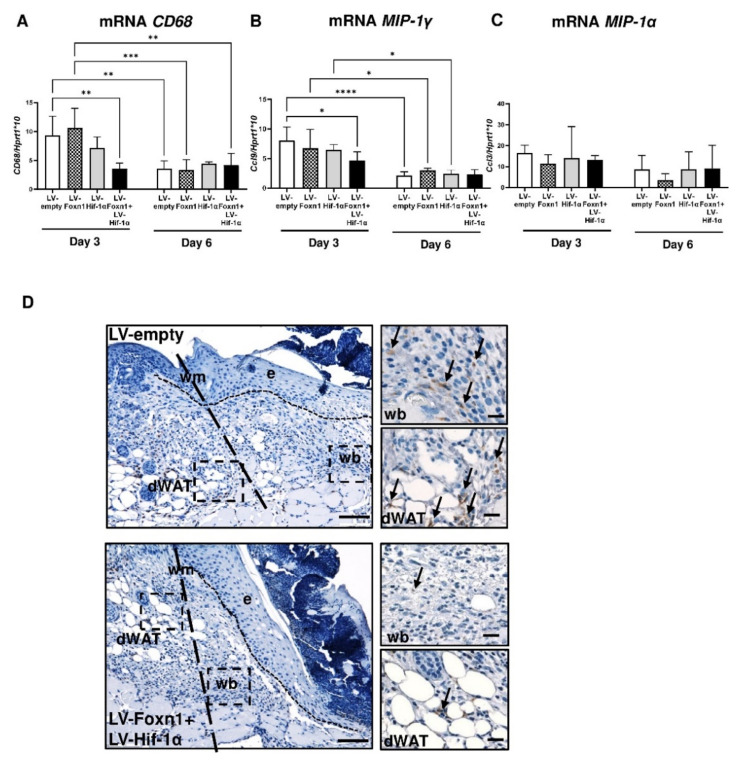
Inflammatory response during skin wound healing in LV-empty, LV-Hif-1α, LV-Foxn1- or LV-Hif-1α+LV-Foxn1-injected Foxn1^−/−^ mice. *CD68* (**A**), *MIP-1γ* (**B**) and *MIP-1α* (**C**) mRNA expression analysis (*n* = 4 mice per group/per time point). (**D**) Representative histological sections of wounded LV-empty or LV-Hif-1α+LV-Foxn1-injected Foxn1^−/−^ mice at post-wounding day 3, stained for CD68-positive cells (arrows); wm—wound margin, e—epidermis, wb—wound bed and dWAT—dermal white adipose tissue; scale bar 100 μm, insets 20 µm. Asterisks (**A**–**C**) indicate significant differences (* *p* < 0.05; ** *p* < 0.01; *** *p* < 0.001; **** *p* < 0.0001). Data represents mean ± SD.

The detected reduction in the percentage of CD68 positive cells within LV-Foxn1 treated post-wounded skin (see Figure 2E), prompted us to further analyze the effect of Foxn1/Hif-1α on skin macrophage content. Firstly, we compared the *CD68* mRNA skin expression levels among experimental groups and between post-wounding days: day 3 vs. day 6 (Figure 8A).

Generally, higher levels of *CD68* mRNA expression were detected at post-wounding day 3 in comparison to day 6 (Figure 8A). Mice injected with a combination of LV-Foxn1+LV-Hif-1α showed low (*p* < 0.01), reduced in comparison to control (LV-empty) mice levels of *CD68* mRNA expression at post-wounding day 3 (Figure 8A). There were no differences in *CD68* among treatment groups, at post-wounding day 6. This results were further supported by the analysis of macrophage inflammatory protein *-1*: *MIP-1γ* (Ccl9; Figure 5B) and *MIP-1α* (Ccl3; Figure 5C) mRNA expression levels. The analysis of *MIP-1γ* mRNA expression reflects the results obtained for *CD68* (compare Figure 8A,B). At post-wounding day 3, the lowest and reduced levels of *MIP-1γ* mRNA expression, in comparison to LV-empty treated mice, were detected in the group treated with a combination of LV-Foxn1+LV-Hif-1α (Figure 8B). No differences in *MIP-1α* mRNA levels of expression were detected among treatment groups or post-wounding days 3 vs. 6 (Figure 8C). To test if Foxn1 and/or Hif-1α regulates the localized recruitment of macrophages to the post-wounded skin of Foxn1^−/−^ mice, we examined the distribution of CD68 positive cells in sectioned wounded skin (Figure 8D). At post-wounding day 3, CD68 positive cells were detected in the dermal part of the skin, particularly at the wound margin and wound bed, and among the adipocytes forming dWAT (Figure 8D). However, we observed the differences in CD68-positive cell accumulation among the experimental groups. Immunostaining revealed abundant CD68 positivity in the control (LV-empty) treated skin of Foxn1^−/−^ mice, and decreased to a few CD68-positive cells in the skin of LV-Foxn1+LV-Hif-1α treated mice (Figure 8D).

Overall, the data indicate that Foxn1, together with Hif-1α, decrease the macrophage skin content during the early (day 3) stage of wound healing.

## 3. Discussion

The results of the present in vivo study can be viewed in terms of three separate aspects of skin biology: (1) the role of Foxn1 and Hif-1α in the regulation of dWAT in wounded skin through the Igf2 signaling pathway; (2) the control of the macrophages content in post-wounded skin, which is synchronized by Foxn1 and Hif-1α and (3) LV delivery as an efficient and valuable method to study the effects of Foxn1 and Hif-1α on skin biology. We demonstrated that the delivery of Foxn1 and Hif-1α into the wounded skin of Foxn1^−/−^ mice, whose skin is characterized by low levels of Hif-1α expression, modifies dWAT. These data are the first to document that Foxn1 and Hif-1α cooperatively regulate dWAT during the proliferative phase of skin wound healing through the Igf2 signaling pathway.

The induction of Hif-1α in the preadipocytes and adipocytes of white fat depots, has been documented in vitro and in vivo, in addition to the impact of hypoxia on adipose tissue functionality [35,36,37,38]. Similarly, hypoxia signaling, including Hif-1α, in the regulation of wound healing and fibrosis has attracted a large number of studies, which have been compiled in many reviews [32,33,34]. However, to our knowledge, the possible role of Hif-1α and Foxn1 in dWAT regulation during the wound healing process has not yet been explored.

Our previous investigations showed that Foxn1^−/−^ mice displayed impairments in the regulation of Hif-1α during the skin wound healing process [39]. We also demonstrated that a decrease in Foxn1 activity is related to a resistance to diet-induced obesity in Foxn1^+/−^ mice, and that Foxn1 regulates dWAT capacity since Foxn1^+/−^ mice showed an altered expression of adipogenic genes in non-injured and post-injured skin [12]. Moreover, growth factors Igf2 and Bmp2, members of the adipogenic signaling pathway, were gradually downregulated, along with Foxn1 deactivation in the skin of Foxn1^+/+^, Foxn1^+/−^ and Foxn1^−/−^ mice [12].

In the present study, we focused on the early proliferative phase of skin wound healing (days 3 and 6 after wounding) and the role of Foxn1 and Hif-1α in dWAT regulation. The analysis of adipocyte number, size and diameter was performed in two separated areas of wounded skin: the wound edge (the “old”, pre-wounded skin tissues) and wound bed (the “new”, post-wounding restored tissues). Adipocyte parameters analyzed at the wound edge showed a decrease in adipocyte size and diameter between days 3 and 6, particularly for the skin treated with the Foxn1 and Hif-1α transgenes. Shook et al. detected a decrease in adipocyte size, but not in the number in the wounded area, which was further confirmed by lipidomic and genetic experiments, revealing that lipolysis adjacent to wound adipocytes by 3 days after injury stimulates an inflammatory response attracting macrophages, which consequently stimulates the repair process [19]. In our study, we did not identify any changes in lipolytic gene *Atgl* expression; however, the examination was performed on post-wounding days 3 and 6. Intriguingly, in contrast, on post-wounding day 6, we detected the increase in the levels of *Fasn* and *Glut4* mRNA expression, the genes related to lipogenesis (see Figure 7B,D). The increase was identified exclusively in the skin treated with a combination of both transgenes, Foxn1 and Hif-1α, at post-wounding day 6, but not day 3. Considering that Glut4 is a major insulin-regulated glucose transporter expressed in white adipose tissues [44], its increase, together with the increase in *Fasn* gene expression, can indicate the first steps in lipogenic dWAT reconstruction in post-wounded skin that is stimulated by Foxn1/Hif-1α signaling. We also observed that the wounded skin of Foxn1-, Hif-1α- and Foxn1+Hif-1α-transduced skin, but not control skin, showed the robust upregulation of *Igf2* on post-wounding day 6. Interestingly, a simultaneous increase in *Igf2*, *Glut4* and *Fasn* in mouse post-wounded skin detected in our study was also observed in human subcutaneous skin, but not in visceral adipocytes [45]. Whereas, IGF2 promoted the differentiation of human subcutaneous preadipocytes, it decreased the differentiation of the visceral one. Moreover, the differentiated subcutaneous adipocytes stimulated by IGF2 showed an abundant increase in GLUT4 and FASN protein, while visceral adipocytes treated by the same dose of IGF2 displayed the reduction of both GLUT4 and FASN [45]. Our data support the subcutaneous adipocytes-specific pattern in IGF2, GLUT4 and FASN upregulation [45], detected by Alfares et al. Moreover, our data also point out the possible cooperative role for Foxn1/Hif-1α in Igf2, Glut4 and Fasn pathway regulation. Considering that Igf2 has been recognized as a fetal growth promoter, it is conceivable that reconstruction of post-wounded skin that, in numerous aspects, recapitulates embryonic skin development [46], can involve the Foxn1/Hif-1α/Igf2 pathway.

Among the Hif-1α, Foxn1, Hif-1α+Foxn1 or control groups in our study, the most prominent differences were detected in the wound bed in newly formed tissues. Histological and adipogenic gene expression analyses showed that the presence of both Hif-1α and Foxn1 regulates dWAT. Whereas the wounded skin of Foxn1^−/−^ mice (control) displayed the presence of adipocytes at the wound bed, treatment with Foxn1, together with Hif-1α, but not separately, resulted in dWAT absence/reduction. The lack of adipocyte precursor cells in wounded skin during the proliferative phase of healing was observed by Plikus et al. [23]. Instead, the appearance of myofibroblasts at day 5 post-wounding, which became largely abundant in the post-wounding tissues at day 12, was detected [23]. The first signals of adipocyte appearance marked by *Zfp423* and *Cebpβ* expression, were detected in the cells adjacent to new hair follicles in wounded skin on days 21–24. Further experiments revealed that myofibroblasts adjacent to hair follicles convert to adipogenic precursors (dWAT formation) [23]. The localization of newly formed adipocytes adjacent to hair follicles, the increase in BMP signaling, the temporal increase in *Zfp423* and the transcription factor driving of mesenchymal progenitors into the adipolineage, on post-wounding days 21–24, led to the conclusion that wounding stimulates a regenerative pathway to restore dWAT. The authors stress that the process takes place in a particular “window”, when new hair follicles appear in the wound bed (days 21–24) [23]. The stimulatory effect of Foxn1 on BMP2 signaling was observed in our previous in vitro study [9], but not in the present in vivo setting. Neither Foxn1 alone nor in combination with Hif-1α, stimulated Bmp2 expression in wounded skin at days 3 or 6. This can be related to the post-wounding timing; an increase in Bmp2 expression was observed on days 21–24 when hair follicles regenerated at the wound bed [23]. Considering that Foxn1 is expressed in hair follicles and its involvement, together with Bmp2, in hair growth is evident [47], in our present study, the lack of Bmp2 upregulation on post-wounding day 6, seems to be related to the early period of wound healing when hair follicle regeneration does not take place.

Another aspect of the present data is the role of Foxn1 in the regulation of macrophages content in post-wounded skin. Although intensely studied, the exact impact of macrophages on skin wound healing is still under debate/exploration [48,49]. In the present study, we showed that Foxn1 and Hif-1α together decreased levels of *CD68* and *MIP-1γ* mRNA expression, decreased the percentage of CD68 positive cells and reduced side-specific localization of CD68 positive cells in the wounded skin of Foxn1^−/−^ mice (see Figure 8). MIP-1γ is a chemokine that induces chemotaxis of macrophages [50]. The similar pattern detected in the reduction levels of *MIP-1γ* and *CD68* mRNA expression, particularly at post-wounding day 3, can indicate the role of the Foxn1/Hif-1α transgenes in the resolution of the inflammatory phase of the healing. The data are in agreement with our previous study that showed the extensive accumulation of CD68-positive cells and higher levels of monocyte chemoattractant protein 1 (MCP-1) in the wounded skin of Foxn1^−/−^ mice [39]. Whether Foxn1/Hif-1α, through macrophages content regulation, can contribute to skin wound healing, remains an important issue for future research.

We also showed that LVs carrying transgenes are an efficient method of delivering transgenes of interest, i.e., Foxn1 and Hif-1α, into the skin. Our study confirmed the effectiveness of the LV intradermal gene in in vivo delivery [40,41,42]. However, instead of the immunohistological assessment [40] or bioluminescence imaging [42] of LV gene delivery, we applied a flow cytometry assay to validate the efficiency of LV transgene incorporation by skin cells, which allowed us not only to estimate the percentage of transduced skin cells. but to also characterize the phenotype of those cells. The characteristics of the cells carrying LV-Hif-1α and/or LV-Foxn1 are in agreement with their native cell expression. The high percentage of E-cad/Vim cells in comparison to E-cad alone, and the high percentage of CD68-positive cells within LV-Hif-1α-carrying transgenes, indicate that Hif-1α was incorporated into keratinocytes, dermal fibroblasts and macrophages [31,46,47]. LV-Foxn1-carrying cells were positive for CK6 or vimentin. Since Foxn1 localization in the skin is limited to the epidermis, particularly to the cells that are activated by skin wounding (CK6 positive), the relatively high levels in the vimentin population are unexpected. Our previous study performed on transgenic Foxn1:Egfp mice, documented the involvement of Foxn1 in the EMT process [27,28]. We detected the presence of more than 20% of Foxn1/E-cadherin/N-cadherin triple-positive cells exclusively in the skin of newborn Foxn1:Egfp mice, at the period of life when the EMT process occurred [28,48]. We also previously showed an increase in Foxn1/E-cadherin/N-cadherin- and Foxn1/N-cadherin-positive cells, and cells that localize Foxn1 with Snail1, a marker of EMT, in the wounded skin of Foxn1:Egfp mice, on days 5–6 [27]. Therefore, the relatively high percentage of vimentin-positive cells among LV-Foxn1-transduced skin cells, which increased particularly at day 6 post-wounding, confirms our previous data.

In summary, these data are the first to highlight the role of Foxn1 and Hif-1α in dWAT regulation in wounded mouse skin. We also showed the molecular cross-talk between Foxn1 and Hif-1α in in vivo settings, which confirmed our previous in vitro data [39]. We propose that evoked by hypoxia upregulation of Foxn1 and Hif-1α regulates skin wound healing through: (1) the stimulation of Igf2 signaling; (2) increasing expression levels of *Glut4* and *Fasn*, the genes involved in lipogenesis, and (3) reduction in macrophages content in the wound site.

## 4. Materials and Methods

### 4.1. Lentiviral Vector Construct

Lentiviral transfer vectors were prepared by cloning full-length Hif-1α (NM_001313919) into pEZ-Lv215, to generate Hif-1α-IRES-GFP, and full-length FOXn1 (NM_008238) into pEZ-Lv214, to produce FOXn1-IRES-Cherry (GeneCopoeia, Rockville, MD, USA). Lentivirus particles containing either transgenes or just eGFP (empty/control), were produced in the 293FT packaging cell line (high-glucose DMEM, 10% FBS, 0.1 mM NEAA, 1 mM MEM sodium pyruvate, 6 mM L-glutamine, 1% penicillin/streptomycin and 0.5 mg/mL geneticin) by transient cotransfection using the calcium–phosphate precipitation method [51]. Briefly, 40–60% confluent HEK 293FT cells plated on 10 cm dishes 24 h earlier, were transfected with 14 μg of transfer vector, 4 μg of packaging vector psPAX2 (gift from Didier Trono (Addgene plasmid # 12260, Watertown, MA, USA)), and 2 μg of envelope vector pMD2.G (gift from Didier Trono (Addgene plasmid # 12259, Watertown, MA, USA)) and mixed in 0.45 mL of water, 2.5 M CaCl_2_, and 2x HeBS (274 mM NaCl, 10 mM KCl, 1.4 mM Na2HPO4, 15 mM D-glucose, 42 mM HEPES, pH 7.06) per plate. Before adding to the cells, the DNA–HeBS mix was incubated for 30 min at RT. After 16 h of incubation, the medium with the DNA precipitate was gently removed from the cells and replaced fully with fresh medium. Medium containing viral particles was collected after 72 h, spun at 300 ×g for 5 min at room temperature to remove cell debris, filtered through a 0.45 µm PES filter, and concentrated by ultracentrifugation for 2 h at 26,000× *g* and 4 °C (rotor SW-32Ti, Beckman Coulter, Brea, CA, USA). The supernatant was carefully removed, and the remaining medium was removed by centrifugation at 180× *g* for 2 min. The pellet containing lentiviral particles was suspended in PBS for 2 h with occasional gentle vortexing and stored at +4 °C otherwise. The lentiviral suspension was divided into aliquots, snap-frozen and stored at −70 °C. The functional titer was measured in 293FT cells using a BD LSRFortessa cell analyzer flow cytometer (BD LSRFortessa, Becton Dickinson, Franklin Lakes, NJ, USA).

### 4.2. Animals and Experimental Protocol

#### 4.2.1. Animal Studies

For animal studies, 7 to 12 weeks old Foxn1^−/−^ (adult nude mice; CBy. Cg-Foxn1 <nu>/cmdb; *n* = 32) and Foxn1^+/+^ (Balb/c/cmdb) mice of a similar age (*n* = 10) were used. Mice were housed in individually ventilated cages (IVCs), in a temperature- and humidity-controlled room (22 °C and 55%, respectively) with a 12 h light/12 h dark cycle, at the Center of Experimental Medicine (CEM), Medical University of Bialystok, Poland. The mice and the wounded and LV-injected skin areas were observed every post-wounding day, with meticulous, detailed notes made by an unbiased experimenter. All experimental animal procedures were approved by the Ethics Committee of the University of Warmia and Mazury (Olsztyn, Poland), No. 68/2018.

#### 4.2.2. Wound Model and Surgical Procedure

The day before the wounding procedure (day -1), Balb/c mice were anesthetized, the hair was shaved in the dorsal area, and the skin was cleaned with Octenisept (Schulke & Mayer, Norderstedt, Germany). The next day (day 0), Foxn1^−/−^ and Balb/c (Foxn1^+/+^) mice were anesthetized with isoflurane. Using a 4 mm dermal biopsy punch (Miltex, GmbH, Rietheim-Weilheim, Germany), four excisional skin wounds were created on the dorsal skin (Figure 1). After wounding, mice were transferred to individual cages and observed until recovery. The following day (day 1), Foxn1^−/−^ mice were anesthetized with isoflurane. Lentivirus vectors (LVs) expressing the Foxn1 or Hif-1α gene or LV-empty (control vector carrying eGFP), were thawed and diluted in phosphate-buffered saline (PBS) (Gibco, Thermo Fisher Scientific, Waltham, MA, USA) to obtain a dose of 10^6^ TU in a 50 µL volume. LVs were intradermally injected into the base and margin of each wound on the dorsal skin of the mice, keeping the injection evenly distributed (Figure 1). At days 3 and 6, after wounding, the animals were sacrificed. Post-wounded skin tissues (*n* = 4 animals/per vector/per day of wounding) were collected post mortem, with an 8 mm biopsy punch and stored in liquid nitrogen for RNA isolation (*n* = 4 per group) or fixed in 10% formalin (*n* = 3 per group) for histological analysis. In addition, cells isolated from enzymatically digested wounded tissue (*n* = 4 per group/per day) were analyzed by flow cytometry (Figure 1).

### 4.3. Microscopic Analysis of Wounded Skin

Formalin-fixed post-wounding skin tissues were embedded in paraffin and sectioned at 5 µm thickness. The immunohistochemical detection of cytokeratin 16 (CK16, 1:300; Cat# b7609, LsBio, Seattle, WA, USA) and perilipin 1 (PLIN1, 1:200; ab3526, Abcam Cambridge, MA, USA) (Table S1) was performed using the protocol previously described [27]. Briefly, slides were deparaffinized, rehydrated and heated in citrate buffer for antigen retrieval. Antibody binding was detected with the ABC complex (Vectastain ABC Kit, Vector Laboratories, Inc., Burlingame, CA, USA). Peroxidase activity was revealed using 3,3-diaminobenzidine (3,3-DAB) (Sigma-Aldrich, St. Louis, MO, USA) as a substrate. Slides were counterstained with hematoxylin, visualized using an Olympus microscope (BX43) and analyzed with Olympus CellSens Software (Olympus, Tokyo, Japan).

The re-epithelialization process in the wounded skin of mice was assessed, microscopically, on CK16-stained histological slides, according to a previously described protocol [52]. The percentage of re-epithelialization was calculated by the following formula: (length of the extending epidermal tongues)/(length of the wound) × 100% (number of mice *n* = 2–3 per group/per day). Measurements of adipocyte number, size and diameter in the dWAT layer were performed on PLIN1-stained skin sections (*n* = 3 mice per group), using ImageJ software version v1.53k (National Institutes of Health, Bethesda, MD, USA), as described previously [9]. To measure the adipocyte size, two methods were applied: (1) the measurement of adipocyte size as a cell area (volume) and (2) feret diameter as a measure of a cell size along a specified direction (the longest distance between any two points restricting the object perpendicular to that direction) [53,54].

### 4.4. RNA Isolation and Quantitative RT-PCR

Total RNA was extracted from skin samples using the TRIzol Reagent (Thermo Fisher Scientific, Waltham, MA, USA), according to the manufacturer’s instructions. The quantity of RNA was verified on a NanoDrop 1000 (Thermo Fisher Scientific, Waltham, MA, USA). The quality of RNA was analyzed by performing agarose gel electrophoresis. The cDNA was synthesized from 500 ng of total RNA using a High-Capacity cDNA Reverse Transcription Kit with RNase Inhibitor (Thermo Fisher Scientific, Waltham, MA, USA). To measure the mRNA expression of *Hprt1*; *mCherry*; *eGFP*, *Pparγ*; *Fapb4*; *Mest*; *Zfp 423*; *Igf2*; *Bmp2*; *Srebp1c*; *Fasn*; *Glut1*; *Glut4* and *Atgl*, Single Tube TaqMan^®^ Gene Expression Assays (Thermo Fisher Scientific, Waltham, MA, USA) were used (Table S2). *Hprt1* was chosen as the most stable housekeeping gene during cutaneous wound healing, after an analysis described previously [28]. Amplification was performed using a 7900HT Fast Real-Time PCR System (Applied Biosystems by Thermo Fisher Scientific, Waltham, MA, USA) under the following conditions: initial denaturation for 10 min at 95 °C, followed by 40 cycles of 15 sec at 95 °C and 1 min at 60 °C. Each run included a standard curve based on aliquots of pooled skin sample RNA. All samples were analyzed in duplicate. The mRNA expression levels were normalized to the reference gene *Hprt1* and multiplied by 10.

### 4.5. Flow Cytometry Analysis

Flow cytometry was performed on wounded skin collected from Foxn1^−/−^ mice injected with LVs expressing Foxn1-mCherry or Hif1α-eGFP, or a mix of Foxn1-mCherry and Hif1α-eGFP or control vector LV-eGFP (empty). Skin samples were collected 3 and 6 days after wounding and processed, as previously described [39]. In brief, skin tissues were washed in 70% ethanol and PBS, minced and enzymatically digested in 3.68 mg/mL collagenase (Sigma-Aldrich Co., St. Louis, MO, USA) for 80 min. Following a series of centrifugations at 270× *g* for 5 min, cells were filtered through 100 μm cell strainers (Falcon, A Corning Brand, NY, USA) and counted with a hemocytometer (Countess II, Thermo Fisher Scientific, Waltham, MA, USA). For multiple staining, cells were incubated with the following antibodies: anti-mCherry-Alexa Fluor 647 (Thermo Fisher Scientific, cat # M11241, Waltham, MA, USA); anti-GFP-Alexa Fluor 488 (Thermo Fisher Scientific; cat # A21311, Waltham, MA, USA); CD45.2-PE (BD Pharmingen, cat # 560695); anti-E-cadherin-APC (BioLegend, cat # 147312, San Diego, CA, USA); anti-CD68—PE (BD Pharmingen, cat # 566387, San Diego, CA, USA); anti-keratin 6 (NSJ Bioreagents, cat # V2168) and anti-vimentin (Abcam, cat # ab92547, Cambridge, UK), conjugated with FITC (Lynx Rapid FITC Antibody Conjugation Kit, cat # LNK061F; Bio-Rad Laboratories, Inc., Berkeley, CA, USA) and PeCy7 (Lynx Rapid PECY7 Antibody Conjugation Kit, cat # LNK112PECY7 (Table S1). Labeled cells were washed in a perm/wash buffer (BD Biosciences, Franklin Lakes, NJ, USA) and fixed in a cytofix/cytoperm buffer (BD Biosciences, Franklin Lakes, NJ, USA). A BD LSR Fortessa Cell Analyzer flow cytometer (Becton Dickinson and Company, BD Biosciences, San Jose, CA, USA) and BD FACS Diva v6.2 Software (Becton Dickinson, Franklin Lakes, NJ, USA) were used. The total number of cells counted for each sample was ~50,000.

### 4.6. Statistical Analysis

Statistical analysis was performed with GraphPad Prism, Version 9.1.2 (GraphPad Software, La Jolla, CA, USA). All data were checked for normality using the Shapiro–Wilk test. One-way analysis of variance with post hoc Tukey’s test or two-way analysis of variance were used. Data are expressed as the mean ± standard deviation (SD). A value of *p* < 0.05 was considered statistically significant; * *p* < 0.05, ** *p* < 0.01, *** *p* < 0.001 and **** *p* < 0.0001.

## Data Availability

The data presented in this study are available in the article and in supplementary material here.

## Supplementary Materials

The following are available online at www.mdpi.com/article/10.3390/ijms23010257/s1.
