# Supplementary material for: Dermal White Adipose Tissue (dWAT) Is Regulated by Foxn1 and Hif-1α during the Early Phase of Skin Wound Healing
Source: Int J Mol Sci. 2021 Dec 27;23(1):257. doi: 10.3390/ijms23010257 (PMC8745105; doi:10.3390/ijms23010257)
Supplement: Supplementary file 1 [file ijms-23-00257-s001.zip › Supp. figures_17.12.21.pptx]

## Slide 1
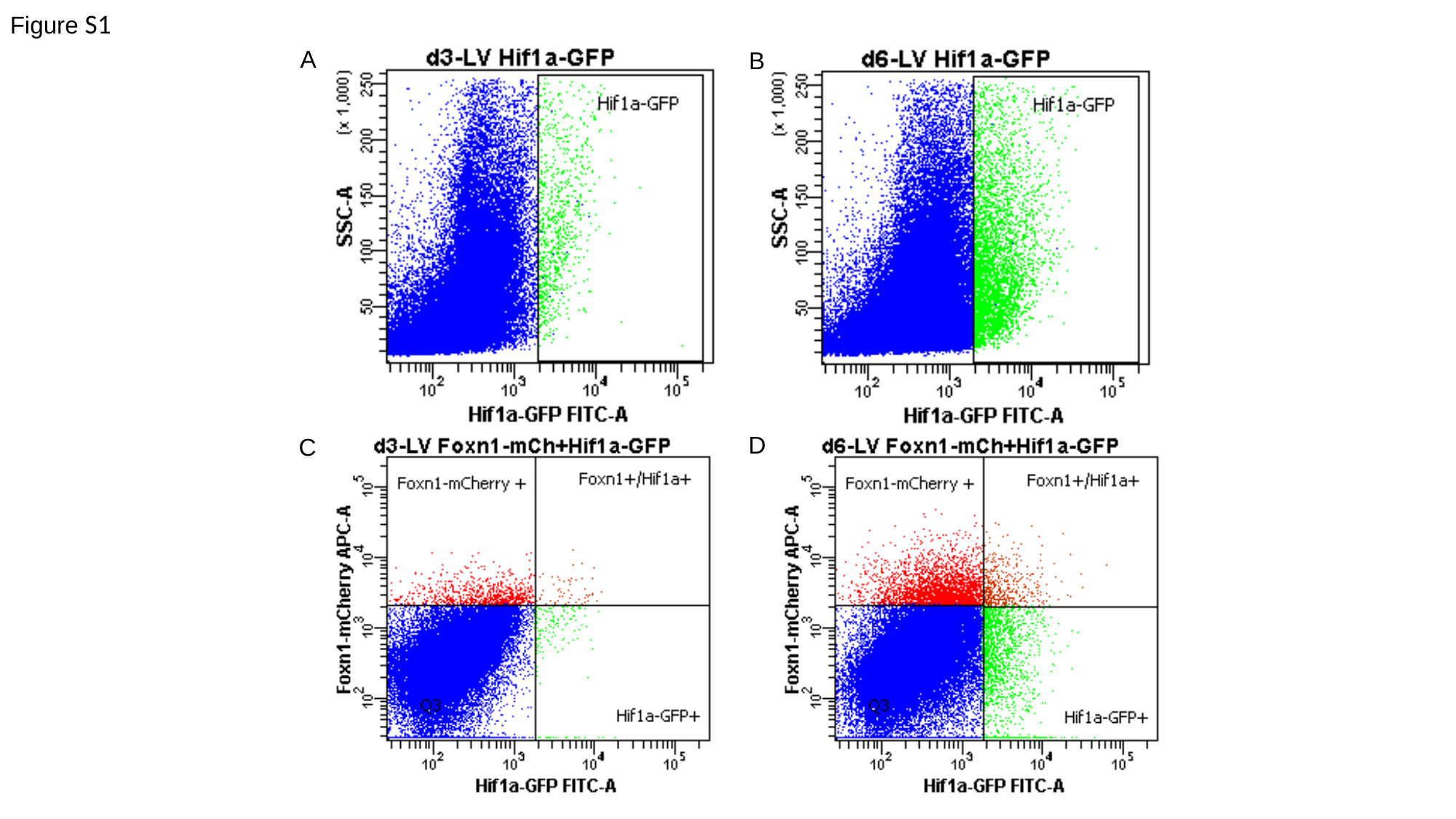

Figure S1
A
B
D
C

## Slide 2
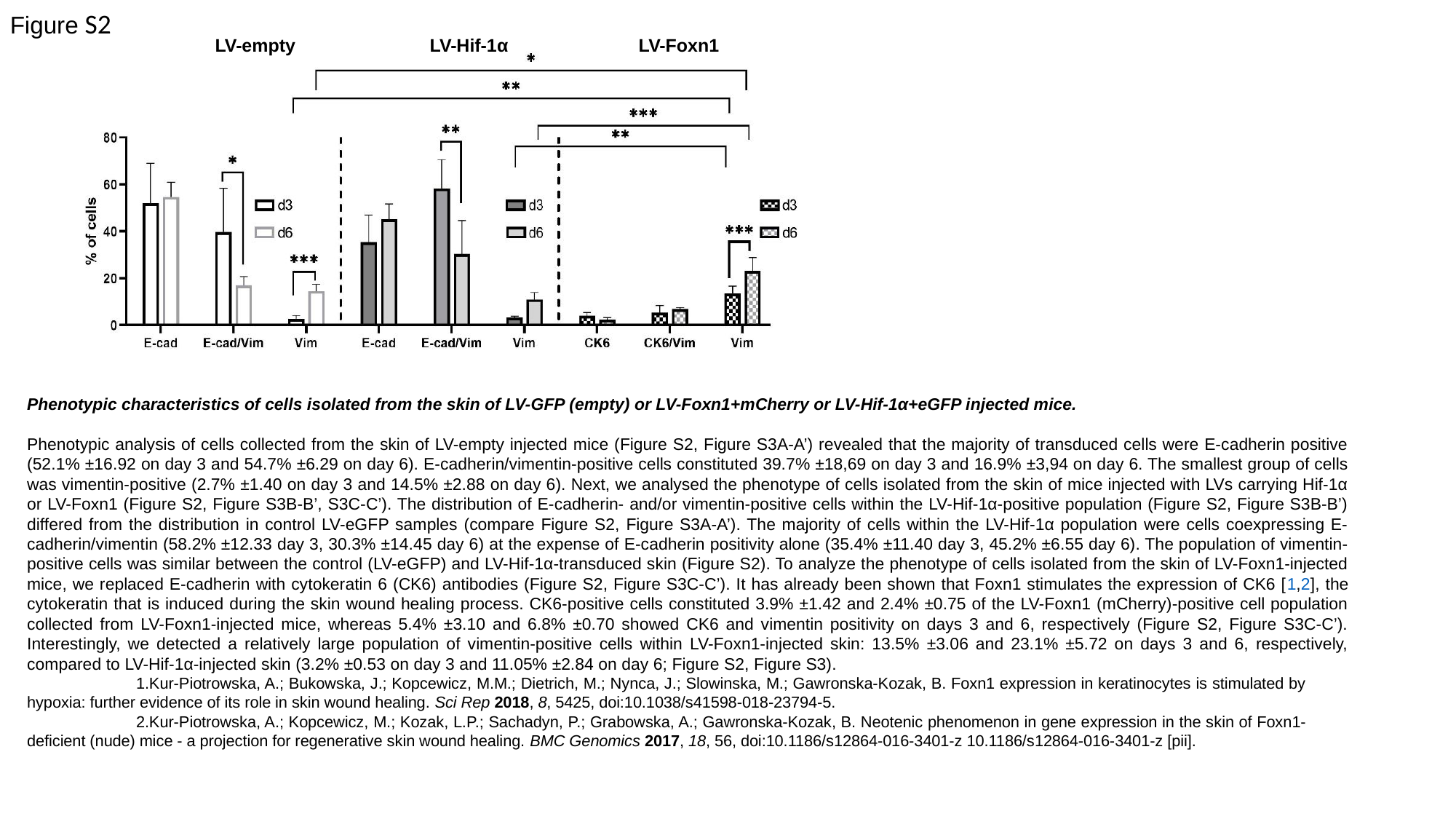

Figure S2
LV-empty
LV-Hif-1α
LV-Foxn1
Phenotypic characteristics of cells isolated from the skin of LV-GFP (empty) or LV-Foxn1+mCherry or LV-Hif-1α+eGFP injected mice.
Phenotypic analysis of cells collected from the skin of LV-empty injected mice (Figure S2, Figure S3A-A’) revealed that the majority of transduced cells were E-cadherin positive (52.1% ±16.92 on day 3 and 54.7% ±6.29 on day 6). E-cadherin/vimentin-positive cells constituted 39.7% ±18,69 on day 3 and 16.9% ±3,94 on day 6. The smallest group of cells was vimentin-positive (2.7% ±1.40 on day 3 and 14.5% ±2.88 on day 6). Next, we analysed the phenotype of cells isolated from the skin of mice injected with LVs carrying Hif-1α or LV-Foxn1 (Figure S2, Figure S3B-B’, S3C-C’). The distribution of E-cadherin- and/or vimentin-positive cells within the LV-Hif-1α-positive population (Figure S2, Figure S3B-B’) differed from the distribution in control LV-eGFP samples (compare Figure S2, Figure S3A-A’). The majority of cells within the LV-Hif-1α population were cells coexpressing E-cadherin/vimentin (58.2% ±12.33 day 3, 30.3% ±14.45 day 6) at the expense of E-cadherin positivity alone (35.4% ±11.40 day 3, 45.2% ±6.55 day 6). The population of vimentin-positive cells was similar between the control (LV-eGFP) and LV-Hif-1α-transduced skin (Figure S2). To analyze the phenotype of cells isolated from the skin of LV-Foxn1-injected mice, we replaced E-cadherin with cytokeratin 6 (CK6) antibodies (Figure S2, Figure S3C-C’). It has already been shown that Foxn1 stimulates the expression of CK6 [1,2], the cytokeratin that is induced during the skin wound healing process. CK6-positive cells constituted 3.9% ±1.42 and 2.4% ±0.75 of the LV-Foxn1 (mCherry)-positive cell population collected from LV-Foxn1-injected mice, whereas 5.4% ±3.10 and 6.8% ±0.70 showed CK6 and vimentin positivity on days 3 and 6, respectively (Figure S2, Figure S3C-C’). Interestingly, we detected a relatively large population of vimentin-positive cells within LV-Foxn1-injected skin: 13.5% ±3.06 and 23.1% ±5.72 on days 3 and 6, respectively, compared to LV-Hif-1α-injected skin (3.2% ±0.53 on day 3 and 11.05% ±2.84 on day 6; Figure S2, Figure S3).
	1.Kur-Piotrowska, A.; Bukowska, J.; Kopcewicz, M.M.; Dietrich, M.; Nynca, J.; Slowinska, M.; Gawronska-Kozak, B. Foxn1 expression in keratinocytes is stimulated by 	hypoxia: further evidence of its role in skin wound healing. Sci Rep 2018, 8, 5425, doi:10.1038/s41598-018-23794-5.
	2.Kur-Piotrowska, A.; Kopcewicz, M.; Kozak, L.P.; Sachadyn, P.; Grabowska, A.; Gawronska-Kozak, B. Neotenic phenomenon in gene expression in the skin of Foxn1- 	deficient (nude) mice - a projection for regenerative skin wound healing. BMC Genomics 2017, 18, 56, doi:10.1186/s12864-016-3401-z 10.1186/s12864-016-3401-z [pii].

## Slide 3
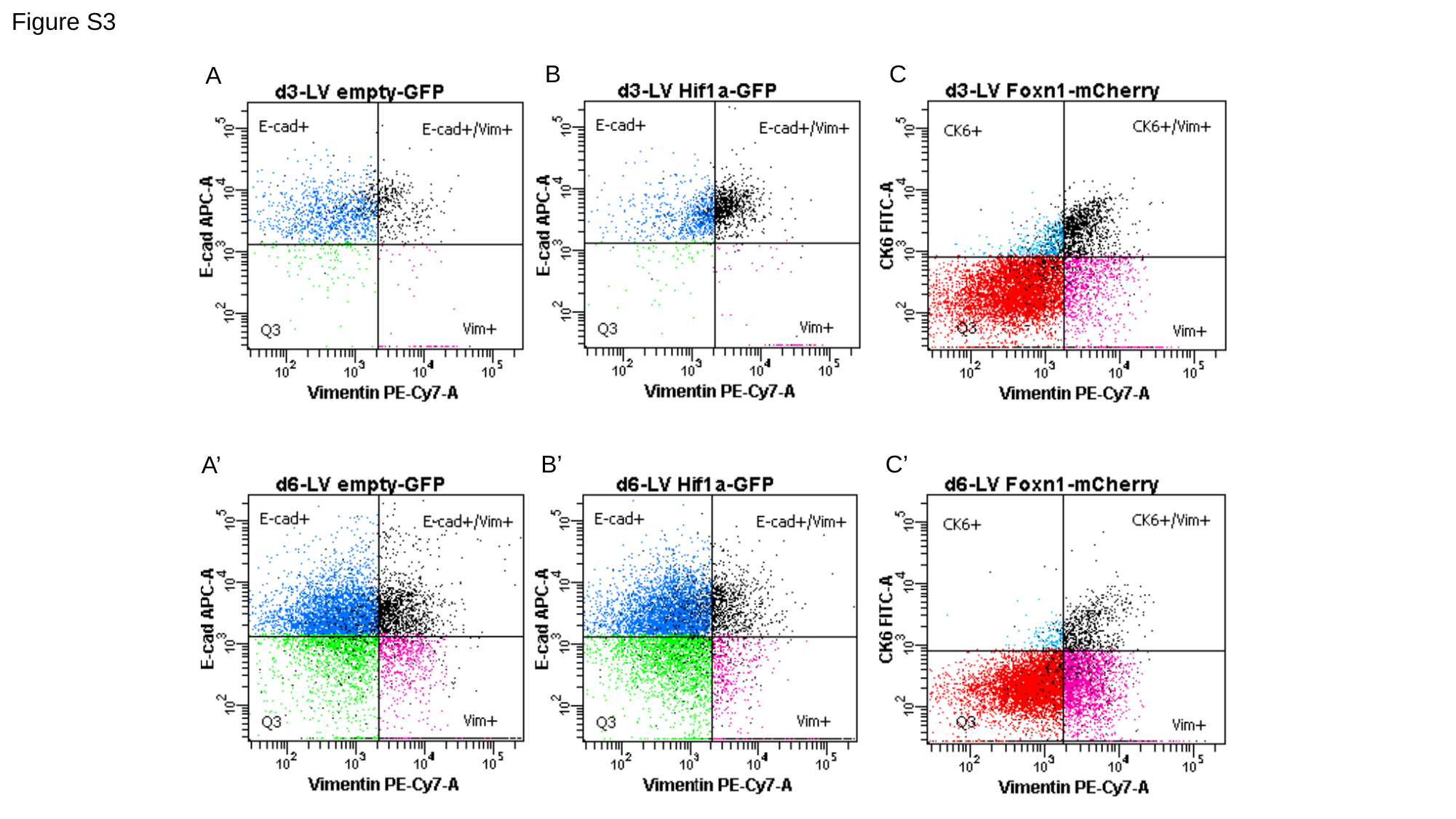

Figure S3
B
C
A
B’
C’
A’

## Slide 4
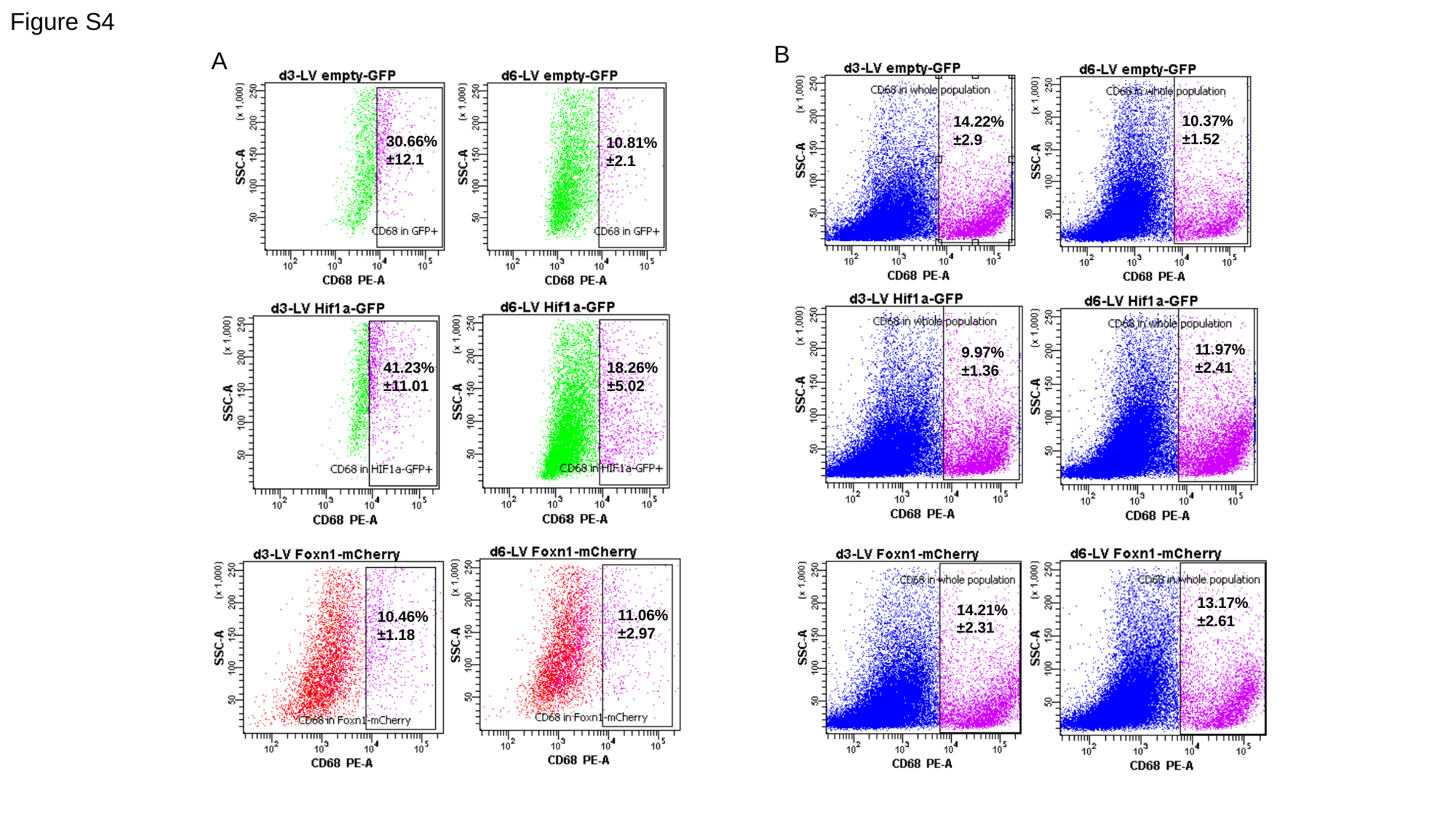

Figure S4
B
A
14.22% ±2.9
10.37% ±1.52
30.66%±12.1
10.81%±2.1
9.97% ±1.36
11.97% ±2.41
41.23% ±11.01
18.26%±5.02
14.21% ±2.31
13.17% ±2.61
11.06% ±2.97
10.46% ±1.18
