# Supplementary material for: Dermal White Adipose Tissue (dWAT) Is Regulated by Foxn1 and Hif-1α during the Early Phase of Skin Wound Healing
Source: Int J Mol Sci. 2021 Dec 27;23(1):257. doi: 10.3390/ijms23010257 (PMC8745105; doi:10.3390/ijms23010257)
Supplement: Supplementary file 1 [file ijms-23-00257-s001.zip › Suppl. Tables_17.12.pdf]

Table S1. List of antibodies used for flow cytometry and immunohistochemistry assays.

Flow cytometry assay				
**Antibody**	**Probe fluorochrome**	**Vendor**	**Cat. No**	**Clone**
CD68	PE	BD Biosciences	566387	FA/11
GFP	Alexa Fluor 488	Thermo Fisher	A21311	
mCherry	Alexa Fluor 647	Thermo Fisher	M11241	16D7
E-cadherin	APC	BioLegend	147312	DECMA-1
Vimentin	PE-Cy7	Abcam	ab92547	EPR3776
Cytokeratin 6	APC	NSJ Boreagents	V2168	LHK6
**Immunohistochemistry**				
**Antibody**	**Concentration**	**Vendor**	**Cat.No**	**Clonality**
Perilipin 1	1:200	Abcam	ab3526	Polyclonal
Cytokeratin 16	1:300	LsBio	b7609	Polyclonal
CD68	1:200	Abcam	ab125212	Polyclonal

Table S2. List of TaqMan gene expression assays

**Gene**	**TaqMan® ID**
Pparɣ	Mm00440940_m1
Mest	Mm00485003_m1
Fabp4	Mm00445878_m1
Zfp 423	Mm00473699_m1
Bmp2	Mm01340178_m1
Igf2	Mm00439564_m1
eGFP	Mr04329676_mr
mCherry	Mr07319438_mr
CD68	Mm03047343_m1
Srebp1c	Mm00550338_m1
Fasn	Mm00662319_m1
Glut1	Mm00441473_m1
Glut4	Mm00436615_m1
Hprt1	Mm01545399_m1
Atgl (Pnpla2)	Mm00503040_m1
MIP-1α (Ccl3)	Mm00441259_g1
MIP-1γ (Ccl9)	Mm00441260_m1
